# P2X3 receptor expression in dorsal horn of spinal cord and pain threshold after estrogen therapy for prevention therapy in neuropathic pain

**DOI:** 10.1016/j.amsu.2020.11.013

**Published:** 2020-11-11

**Authors:** Rohadi Muhammad Rosyidi, Bambang Priyanto, Dewa Putu Wisnu Wardhana, Krisna Tsaniadi Prihastomo, Syauq Hikmi, Agus Turchan, Rozikin

**Affiliations:** a Department of Neurosurgery Medical Faculty of Mataram University, West Nusa Tenggara General Hospital, Mataram, Indonesia; b Department of Neurosurgery, Udayana University Hospital, Medical Faculty of Udayana University, Bali, Indonesia; c Department of Neurosurgery, Dr. Kariadi General Hospital Medical Center, Semarang, Center Java, Indonesia; d Department of Neurosurgery, Dr. R. Koesma General Hospital Medical Center, Tuban, East Java, Indonesia; e Department of Neurosurgery, Dr. Soetomo General Hospital Medical Center, Surabaya, East Java, Indonesia; f Research Unit, Faculty of Medicine, Al Azhar Islamic University, Mataram, Indonesia

**Keywords:** Neuropathic pain, Estrogen, P2X3 receptor, Thermal hypealgesia, Prevention therapy

## Abstract

**Introduction:**

Neuropathic pain may arise from conditions that affecting the central or peripheral nervous system. This study was held to determine the difference P2X3 receptor expression in the dorsal horn of the spinal cord and pain threshold after estrogen therapy in neuropathic pain.

**Methods:**

This study design was an experimental research laboratory. The 24 mice samples divided into negative control group, positive control, and treatment groups. The treatment groups were given subcutaneous injections of estrogen 0.4 ml and also examined for the onset of thermal hyperalgesia in every rat. On day 15, an autopsy was performed on rats, and the spine was taken. The spinal cord was stained by hematoxylin-eosin, and the expression of P2X3 receptors was investigated. P2X3 receptor expression was examined in the dorsal horn on each sample.

**Results:**

From 24 subjects of the study revealed an increase in the onset of thermal hyperalgesia on the estrogen group compared with the placebo group, a higher start. This study also obtained a decrease in the expression of P2X3 on the therapy group compared to the positive control group with significant differences. Statistical test results revealed the appearance of the P2X3 estrogen group had a substantial difference with the placebo group (p = 0.000) and the mean of the negative control group (p = 0.030). The placebo group had a significant difference from the negative control group (p = 0.035).

**Conclusion:**

Estrogen could decrease the expression of P2X3 receptors and prolonged the onset of thermal hyperalgesia. So, both of these explained that estrogen has a role in preventing the occurrence of neuropathic pain after peripheral nerve lesions.

## Highlights

• Experimental study on mice subjects to determine the difference P2X3 receptor expression in the dorsal horn of spinal cord.
• Pain threshold after estrogen therapy in neuropathic pain.
• Estrogen could decrease the expression of P2X3 receptors.
• Estrogen prolonged the onset of thermal hyperalgesia.

## 1 Introduction

At least 40% of all complaints in medical practice is pain. According to the World Health Organization (WHO) data, persistent pain reached 23% of all claims worldwide [1]. Persistent pain represents an important health problem globally and considered on the top ten conditions responsible causes of morbidity [2]. Browsher in 1991 mentions that the incidence of neuropathic pain in the United Kingdom (UK), and the United States reached 1%–2% [3]. The neuropathic pain's illnesses have reached the percentage of at least 27% of all patients in medical practice [4]. The prevalence of neuropathic pain sufferers globally is still unknown, but most studies place an estimate between 1.5% and 8% of people worldwide [5]. The incidence rate of peripheral nerve injury in the United States is about 20 million and annually spent 150$ dollars for health care [4]. The percentage range of chronic pain in spinal cord injuries was from 11 to 94%. About 30% of this chronic pain is a form of neuropathic pain [6].

Tissue damage detected by free nerve endings in peripheral and visceral structures and relayed by primary afferents sensory neurons to second-order sensory neurons in the dorsal horn of the spinal cord and cause pain [5]. Spontaneous sprouting of spared sensory inputs after dorsal root or peripheral nerve lesions was also identified and has been directly associated in development of chronic pain [7].

Neuropathic pain may arise from conditions that affecting the central or peripheral nervous system, which contributes to the pain complex. These changes include the potential for ectopic action, facilitation and disinhibition of synaptic transmissions, loss of synaptic connectivity, and the formation of new synaptic circuits, as well as micro-immunity interactions [8]. Neuropathic pain has a considerable impact on life quality of patients for it causes damage on somatosensory nervous system, which changes its structure and function. Its manifestations, such as dysesthesia, hyperalgesia and allodynia, are regulated by inflammatory mediators that play a role on activation P2X3 receptors [[8], [9], [10]].

ATP is implicated in peripheral pain signaling by action on P2X receptors. P2X receptors are important in disease progression. P2X is a receptors from Dorsal Root Ganglion (DRG) that facilitating pain transmission at peripheral and spinal sites. P2X3 is a subtype of P2X receptors and selectively expressed on the non-peptidergic small diameter sensory neurons. Excitatory of P2X3 and P2X2/3 ATP-gated receptor-channels are thought to exert their effect by directly sensitizing C-fibers by membrane depolarization and calcium entry to facilitate pain transmission [10].

The effect of estrogen in experimental animals will decrease the expression of P2X3 receptors and plays a significant role in preventing and inhibiting the occurrence of neuropathic pain. This study was held to determine the difference P2X3 receptor expression in the dorsal horn of the spinal cord and pain threshold after estrogen therapy in neuropathic pain. This study design was an experimental research laboratory. The 24 mice samples divided into negative control group, positive control, and treatment groups. The treatment groups were administered subcutaneous injections of estrogen 0.4 ml and also examined the onset of thermal hyperalgesia in every rat. On day 15, an autopsy was performed on rats, and the spine was taken. The spinal cord was stained by hematoxylin-eosin, and the expression of P2X3 receptors was examined. P2X3 receptor expression was examined in the dorsal horn on each sample. From 24 subjects of the study revealed an increase in the onset of thermal hyperalgesia on the estrogen group compared with the placebo group, a higher start. This study also obtained a decrease in the expression of P2X3 on the therapy group compared to the positive control group with significant differences. Statistical test results explained that the appearance of the P2X3 estrogen group had a substantial difference with the placebo group (p = 0.000) and the mean of the negative control group (p = 0.030). The placebo group had a significant difference from the negative control group (p = 0.035). Estrogen could decrease the expression of P2X3 receptors and prolonged the onset of thermal hyperalgesia. So, both of these explained that estrogen has a role in preventing the occurrence of neuropathic pain after peripheral nerve lesions. The effects of estrogen are fundamental to the process of homeostasis, cardiovascular regulation, bone metabolism, and inflammatory processes. This hormone acts based on diverse physiological systems inside and outside of the nervous system to make an impact on chronic pain expression, by its binding to the receptors. Estrogen directly affects trial pain. The regulation of estrogen affects neuronal function and pain perception. The effects of estrogen in experimental animals still being debated. In some studies, estrogen has both a pronociceptive effect and an anti-nociceptive effect [11]. Estrogen affects pain modulation through neurotrophin. The low regulation of protein and NGF (Nerve Growth Factor) m-RNA after ovariectomy in mice would return to normal after administration of estradiol. The increased estradiol causes increased expression of NGF receptors on DRG. The provision of soy phytoestrogens can reduce the expression of BDNF (Brain-Derived Neurotrophic) mRNA [12].

Liu and Gintzler proved an increase in the nociceptive threshold in 87% of male rats undergoing orchidectomy with estrogen administration [13]. The mechanism of action of estrogen to modulate pain includes complex mechanisms, one of which is an antinociceptive effect. Estrogen is reported could reduce the sensation of pain, and estrogen that acts on beta receptors can be protected from pain due to inflammation. In neuropathic pain, estrogen can modulate pain through peripheral and central pathways. Estrogen plays a role in the survival and regeneration of spinal neurons. In DRG neurons and spinal horn dorsal horns, estrogen decreases the entry of Ca2+ ions that are triggered by ATP through α estrogen receptors and selectively works to modulate impulses mediated by P2X3 receptors. Centrally, estrogen triggers neurochemical changes that can modulate pain. Estrogen influences the neurotransmission of opiates through mu-opioid receptors. Estrogen is capable of modulating GABAnergic neurons and BDNF expression by neurons [14].

The released ATP through P2X receptor expression in primary afferent neurons occurs in tissue damage and inflammation. The depolarization process will initiate potential action that manifests central pain. One of the P2X receptors, P2X2/3, plays a role in the pain process in chronic inflammation and neuropathic pain. Activation of P2X3 receptors, 2/3 of the receptors of endogenous ATP, contributes to the development of hyperalgesia in inflammation. The P2X receptor is a family of gland-gated ion channels that are activated by ATP extruders that involved in the mechanism of pain [10,15].

Based on the data above and supported by immunohistochemical studies that show that P2X3 receptors are distributed in large quantities in dorsal root ganglia, spinal cord, and brain, the hypothesis of this study is estrogen administration in experimental animals will reduce P2X3 receptor expression and plays a significant role in preventing and inhibiting neuropathic pain. Furthermore, clinical facts show that the use of drugs to treat neuropathic pain such as NSAIDs, narcotics, anticonvulsants, antidepressants, and local anesthetics, or their combination has not yet yielded encouraging results and even these drugs have lost their effectiveness so that breakthroughs are needed to obtain methods to reach the proper management of neuropathic pain.

## 2 Material and methods

This type of research is an experimental study, a completely random design (Completely Randomized Design). The study was conducted at the Pharmacology Laboratory and Anatomical Pathology Laboratory, Faculty of Medicine, Airlangga University/Dr. Soetomo General Hospital, Surabaya.

### 2.1 Animal unit

Wistar white mice outbred strains of albino rats belongs to the species Rattus norvegicus, aged 3–4 months, weighing 150–250 g and in good health. In this study, we tried to weigh 150–250 g of mice, because this study used measurement of thermal hyperalgesia, thereby minimizing the difference in the onset of pain due to heat not due to differences in body weight. The health of experimental animals can be observed from the movements that are quite agile, not lethargic, clean skin and without injury, bright eyes, and not glazed [16,17]. The number of replications used in this study is calculated based on the formula for determining the number of replications to test the hypothesis. The number of replications for each group is obtained by 6.

### 2.2 Correction factors

In anticipation of the experimental unit missing (drop out), a correction factor of 20% is used so that the number of replications per group becomes 7.55–8. So, the total replication is 24 mice. Then the mice were put into a complete randomized treatment group.

### 2.3 Drug treatment

The research subjects were randomly divided into three groups (n = 8 per group): estrogen group, pain threshold value and P2X3 receptor expression (negative control) group, and control (placebo) group. For the estrogen group, the gonad hormone, in the form of estradiol injected subcutaneously on experimental animals, is given by the injection of subcutis (s.c.), which is carried out following routine subcutaneous procedures. Estrogen is given daily for seven days at a dose of 30 μg/kg (0.4 mL/day). Inhibition of hyperalgesia is measured on days 1, 3, 5, 7, and 14 after ligation. For the pain threshold value group, the pain threshold was determined by administering a pain stimulus in the form of a heat stimulus to the algesiameter hotplate.

The negative control group was needed to find out the mice that did not undergo ligation of the sciatic nerve in the ipsilateral foot showed no neuropathic pain when compared to the positive control group. This can be seen from the symptoms by measurement using thermal hyperalgesia, or by supporting examination, which is P2X3 receptor expression. The choice of estrogen in this study was because of the use of estrogen in previous studies to modulate pain [18,19].

Mice pain response is indicated by the reaction of mice in the form of squeaking, licking legs, struggling, or pulling the foot opposite the examined foot (contralateral). Expression of one of the P2X receptor subunits, which are distributed on the surface of the dorsal horn spinal cord; when there is neuronal damage to neuropathic pain. The preparations were taken from the lumbar spinal cord, and immunohistochemistry was examined, and the amount of P2X3 receptor expression was measured in the preparations. For the control group, gender, age, body weight, method of maintenance, neuropathy models, drug dosage, sampling techniques, and sample examination were analyzed.

### 2.4 Number of replication

The number of replications per group was six mice with three treatment groups so that the experimental unit needed was 18 animals. Replication per group was obtained using the Federer formula (1955) and a correction factor of 10% using the Higgins and Klimbaum formulas. Determination of the correction factor of 10%, based on preliminary research because at the end of the preliminary study, no experimental animals were found to drop out or die, and in this study also no experimental animals were found to drop out or die.

### 2.5 Partial sciatic ligation (PSL)

The ipsilateral sciatic nerve binding operation of the PSL model has been performed by opening the muscle, separating and removing the sciatic nerve, then binding ⅓-½ of nerve diameter in the estrogen and positive control group (placebo). In the negative control, group surgery was also performed, but no binding was performed. To determine the success of the neuropathic pain model that was made, then the measurement of rat sensitivity to heat stimulation using a hot plate, 51 °C. Measurements were made on observations of days 1, 3, 5, 7, and 14, then compared between the negative control group and the positive control group.

### 2.6 Hematoxylin-eosin (HE) staining

**Fig. 1:**
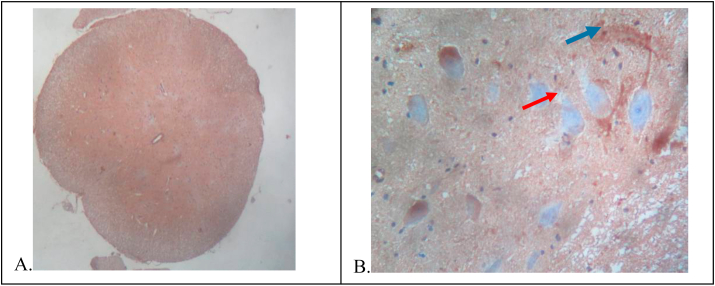
Transverse slices of rat spinal cord with HE staining.

On the 15th day, the rat was euthanasia and surgically obtain spinal cord tissue, and then a histopathological preparation was made with HE staining and IHC preparation with the P2X3 receptor antibody. HE preparations were observed with a 100x magnification light microscope to see the histopathology of the spinal cord and 200x magnification to identify neurons and glia cells and to ensure the feasibility of these preparations for analysis by the IHC technique. A picture of HE preparations in cross-section of the spinal cord of the rat (Fig. 1).

(A) 100× magnification shows the overall histopathology of the spinal cord.
(B) 200× magnification shows a picture of neuron cells (red arrow tip) and glia cells (blue arrow tip).

**Fig. 2:**
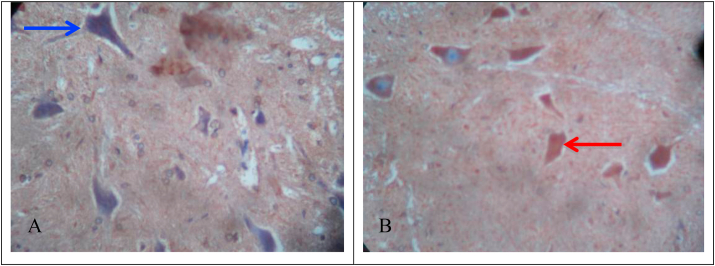
Receptor expression in rat spinal cord (200× magnification).

IHC microscope slide was observed with a 200× magnification light microscope to identify neuron cells that can give a positive reaction to P2×3 receptor antibodies. Positive cells, if cytoplasm and membrane are brown and cells are negative if cytoplasm is clear and the membrane is bluish (Fig. 2).

(A) Negative control group.
(B) Positive control group.

Fig. 2 revealed neuronal cells that give positive reactions (red arrow tip) and those that give negative reactions (blue arrow tip) against P2X3 receptor antibodies.

### 2.7 Statistical analysis

The collected data were analyzed by one-way analysis of variance (ANOVA) tests to determine the potential of estrogen drugs by comparing each estrogen drug with a control group (+). Data is considered normal and homogeneous distribution if the normality and homogeneity test shows p > 0.05, then p > 0.05 data are analyzed by ANOVA. If the data is found to have a value of p < 0.05, the data are considered to be not normally distributed and not homogeneous. The Shapiro-Wilk test data (normality) p < 0.05 was tested with Brown Forsythe. The Levene test data (homogeneity) p < 0.05 was tested by Kruskal Wallis. ANOVA analysis results are said to be meaningful if the value of p < 0.05 is obtained. If the ANOVA analysis results are significant, proceed with the Post Hoc LSD test. Statistical results were performed using the SPSS 16.0. software.

## 3 Results

### 3.1 The effect of estrogen on the thermal onset of hyperalgesia

Measurement of hyperalgesia on days 1, 3, 5, 7, and 14 using a warm plate. Descriptive data were obtained that the onset thermal hyperalgesia on the 1st day with an average of 29.7 s and in the negative control group with an average of 29.8 s. In the positive control group and the estrogen group, there was a decrease in sequential thermal hyperalgesia (Table 1.; Table 2.).

**Table 1:** Test results for normality and homogeneity of hyperalgesia variance on day 1, 3, 5, 7 and 14.

Day	Group	Shapiro-Wilk (p)	Levene test (p)
1	Estrogen	0,411	0132
	Placebo	0,974	
	Negative control	0,905	
3	Estrogen	0,518	0555
	Placebo	0,094	
	Negative control	0,404	
5	Estrogen	0,362	0414
	Placebo	0,020*	
	Negative control	0,505	
7	Estrogen	0,213	0045^
	Placebo	0,479	
	Negative control	0,101	
14	Estrogen	0,874	0595
	Placebo	0,566	
	Negative control	0,492	

The sign (*) indicates the data is not normally distributed (p < 0.05).

The sign (^) indicates inhomogeneous data (p < 0.05).

**Table 2:** The effect of ligation on rat sensitivity to warm plate stimulation. (table based on treatment group).

Group	n	Onset Thermal Hyperalgesia x ± SD (second)				
		Day 1	Day 3	Day 5	Day 7	Day 14
Estrogen group	8	29.71 ± 12.94	28.40 ± 10.35	20.17 ± 11.58	21.05 ± 15.15	9.49 ± 4.76
Placebo group	8	29.81 ± 6.82	22.58 ± 11.48	16.92 ± 7.95	11.07 ± 2.83	11.11 ± 2.83
Negative control	8	29.82 ± 12.75	26.01 ± 13.65	18.03 ± 9.14	15.23 ± 8.58	11.46 ± 5.10
One-way ANOVA*/Brown-Forsythe**/Kruskal-Wallis***		p = 1000*	p = 0,624*	p-0,932***	p = 0,188**	p = 0,633*

The results of normality tests on days 1, 3, 5, 7, and 14 showed that the distribution of data in the 5th-day placebo group was not normal (Shapiro-Wilk test, p < 0.05) so that the analysis of the 5th-day hyperalgesia data using the Kruskal-Wallis test. The variance of data between groups on day seven was found to be not homogeneous (Levene test, p < 0.05) so that the data analysis used Brown-Forsythe statistical analysis and further analysis using the Games-Howell test (Table 1).

Based on Table 2, the results of the analysis comparing between groups, there were no significant differences in hyperalgesia between the estrogen administration group, the placebo group, and the negative control group on each observation day. Data were then analyzed with the same subject ANOVA and paired T-test according to the day of observation in each group (Table 3.) (see Table 4).

**Table 3:** Effect of ligation on mice sensitivity to warm plate stimulation. (table based on day of observation).

Observation day	N	Onset Thermal Hyperalgesia x ± SD (second)		
		Estrogen group	Placebo group	Negative control
Day-1	6	29.71 ± 12.94^ab^	29.81 ± 6.82^a^	29.82 ± 12.75^a^
Day-3	6	28.40 ± 10.35^b^	22.58 ± 11.48^ab^	26.01 ± 13.65^a^
Day-5	6	20.17 ± 11.58^a^	16.92 ± 7.95^bc^	18.03 ± 9.14^ab^
Day-7	6	21.05 ± 15.15^ab^	11.07 ± 2.83^c^	15.23 ± 8.58^bc^
Day-14	6	9.49 ± 4.76^c^	11.11 ± 2.83^c^	11.46 ± 5.10^c^
Same subject ANOVA		p = 0,000	p = 0,002	p = 0,001

Note: a-c: Different superscripts indicate there are differences between groups. (based on the results of paired *t*-test analysis).

**Table 4:** Normality test data result and P2X3 expression homogeneity.

Group	Shapiro-Wilk (p)	Levene test (p)
Estrogen	0,526	0241
Placebo	0,672	
Negative control	0,320

**Table 5:** Descriptive data and P2X3 expression data analysis.

Treatment group	N	P2X3 receptor expression			One-way ANOVA
		‾x ± SD	Minimum	Maximum	
Estrogen group	8	32,19 ± 11,38	12,90	44,83	p = 0,000
Placebo group	8	53,69 ± 7,27	45,00	67,39	
Negative control	7	42,95 ± 7,37	30,43	50,00	

Based on Table 3., the results of the analysis with the same subject ANOVA continued with the paired *t*-test showed there were differences in the onset of thermal hyperalgesia between days of observation in each group. The estrogen group showed significant differences in the onset of hyperalgesia on days 1, 3, 5, and 7 with day 14. In the placebo group, there was a significant difference between day 1 and day 7 and 14. Significant differences were also found between the 3rd day and 7th and 14th-day groups. In negative controls, differences were found on day 1 with day 7 and 14. Also, there are differences between day 3 with day 7 and 14. The onset of hyperalgesia was also found to be significantly different on days 5 and 14.

**Fig. 3:**
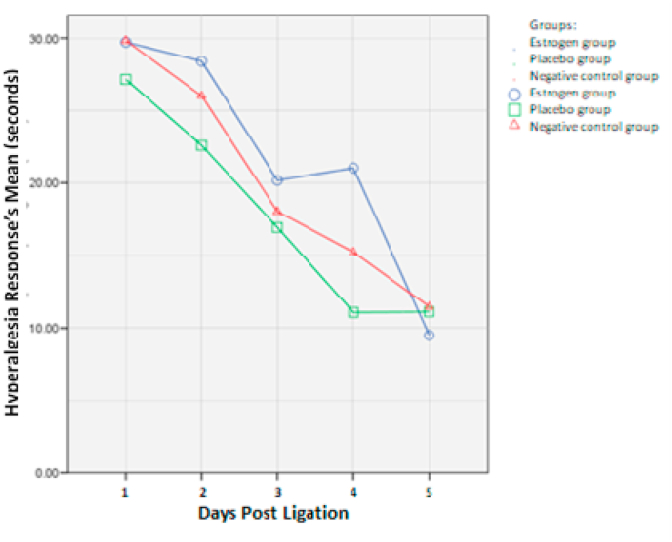
The effect of ligation on mice sensitivity to warm plate stimulus.

**Fig. 4:**
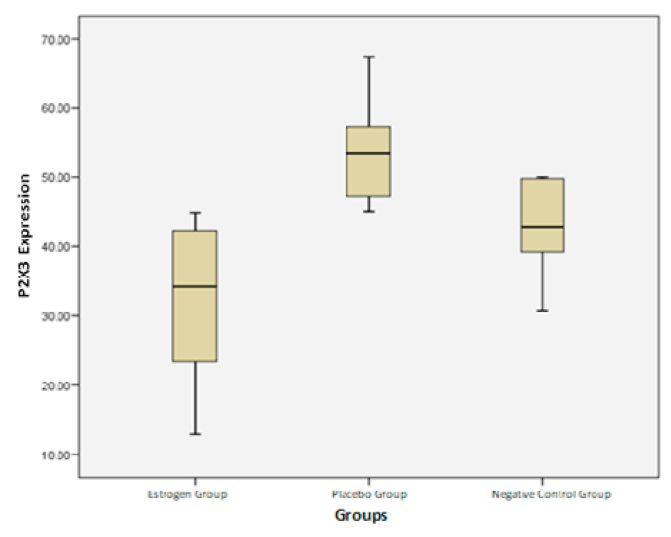
Graph boxplot comparison of P2X3 expressions for each group.

Fig. 3 above explicated, the Estrogen group had a longer onset of hyperalgesia, both compared with the placebo group and the negative control group (see Fig. 4).

### 3.2 Comparison of P2X3 expressions in each treatment group

The P2X3 receptor expression results in each treatment showed that the data were normally distributed from the Shapiro-Wilk test (p > 0.05) and homogeneous from the Levene statistical test (p > 0.05).

Data were then analyzed descriptively and differences in each group were tested with ANOVA which is shown in the following table.

From the comparison boxplot chart of each group, the highest P2X3 expression was found in the placebo group (53.09) and the lowest in the estrogen administration group (32.19).

A comparison of the three groups had significant differences with the ANOVA test (p = 0.001). The data is then continued with the Post Hoc LSD test (Table 6). The Post Hoc LSD test results above showed that the expression of P2X3 in the estrogen group had a significant difference with the placebo group (p = 0,000) and was also significant in the negative control group (p = 0.031). The placebo group had a significant difference from the negative control group (p = 0.031).

**Table 6:** Post Hoc LSD test results.

Group	Estrogen group	Placebo group	Negative control
Estrogen group		0,000	0031
Placebo group			0,031
Negative control			

## 4 Discussion

### 4.1 Effect of PSL on the thermal onset of hyperalgesia

Neuropathic pain is done by making peripheral nerve lesions through ipsilateral sciatic nerve binding. The neuropathic pain model in this study uses the PSL model, which is the binding of the sciatic nerve performed on ⅓ – ½ nerve diameter, so it does not cause paralysis but can cause spontaneous and bilateral pain [20]. Nerve binding is performed using monofilament 8.0 threads, which aim to provide minimal stimulation but can cause neuropathic pain due to nerve lesions. Sciatic nerve binding surgery was performed in the positive control group and estrogen group, meanwhile, in the negative control group no sciatic nerve binding was performed, but the sciatic nerve was also separated and removed from the femoral muscle like the other treatment groups because this study aimed to observe pain caused by nerve lesions so that the entire treatment group experienced nociceptive pain including the negative control group.

Neuropathic pain shows signs and symptoms of hyperalgesia, in the PSL model thermal hyperalgesia symptoms can occur immediately within a few hours to 1 day, then last for more than a minimum of 7 months [21]. So, in this study, hyperalgesia measurement was made 24 h after binding operations and observed every two days to avoid memory factors in mice. Hyperalgesia measurements were carried out using a hot plate with a temperature of 51 °C, and heat exposure continued until there was a reaction from mice in the form of a withdrawal response assessed by the rapid movement (in seconds): hind paw shaking, hind paw licking, or jumping [[21], [22], [23]]. Hyperalgesia measurement is done with two repetitions each, to minimize the subjective factor assessment of pain response.

Fig. 3 Revealed the results of hyperalgesia measurement in each group and observation day. On the first day of observation, the onset of thermal hyperalgesia in the estrogen and placebo groups was not different, but both were above negative control. On the third day of observation, the estrogen group had a higher onset of thermal hyperalgesia compared to the placebo and negative control groups. This happened until the 7th day, where the estrogen group had a higher thermal hyperalgesic onset than the other two groups. However, on the 14th day, the onset of thermal hyperalgesia in the estrogen group was lower than in the placebo group and the negative control group. In Table 3., the effect of ligation on the sensitivity of mice to warm plate stimulation, based on the observation group between the estrogen group, the placebo group, and the negative control group p > 0.05, showed no significant difference in all comparisons, whereas based on Table 5. 3 the effect of ligation on the sensitivity of mice to warm plate stimulation, based on observations of day 1, 3, 5, 7, and 14, it was found that p < 0.05 in all comparisons, showing each difference are significant, it happened because it is a weakness of this study because the number of experimental animals is small and a small frequency of observation (only twice per day of observation).

### 4.2 Effect of estrogen application on thermal onset hyperalgesia of mice

Subcutaneous injection (s.c.) of estrogen is carried out following a routine subcutis procedure. Estrogen is given daily for seven days at a dose of 30 μg/kg (0.4 mL/day).68 Estrogen in previous studies can modulate pain. Estrogen is reported could reduce the sensation of pain, and estrogen that acts on beta receptors can be protected from pain due to inflammation. In neuropathic pain, estrogen can modulate pain through peripheral and central pathways. Estrogen plays a role in the survival and regeneration of spinal neurons. Centrally, estrogen triggers neurochemical changes that can modulate pain. Estrogen influences the neurotransmission of opiates through mu-opioid receptors. Estrogen can modulate GABAergic neurons and BDNF expression by neurons.

Allodynia (pain due to a stimulus that does not usually provoke pain) and hyperalgesia (increased pain from a stimulus that usually provokes pain) are prominent symptoms in patients with neuropathic pain. Neuropathic pain causes signs and symptoms of hyperalgesia, so to understand the inhibition of neuropathic pain in each drug, the measurement of hyperalgesia is performed. Measurement of hyperalgesia on days 1, 3, 5, 7, and 14 using a warm plate. Measurement of hyperalgesia was done 30 min post-injection, each injection and measurement of hyperalgesia were completed per group. It is intended that each group has the same interval between subcutaneous injection with hyperalgesia measurement and 30 min is the average drug can reach peak effect so that it can be seen the pain reduction response between the positive control group and the estrogen group.

Based on the analysis, there was an increase in thermal hyperalgesia onset in the estrogen group when compared with the placebo group, having a higher onset (Table 3, Fig. 3). This shows that estrogen can inhibit neuropathic pain by influencing the P2X3 receptor ion and modulating pain in the peripheral pathway, but in the analysis of different tests, no significant results were obtained. This can be due to the lack of sample size, as well as the lack of onset measurement of hyperalgesia. In this study, a single dose of estrogen injection was performed in mice. Estrogen has a preventive effect by modulating neuropathic pain, but several studies have suggested that estrogen also has a pro-inflammatory effect. The weaknesses of this study are the administration of single-dose drugs, there is no variation of dosages, and the method of administration of drugs is only through subcutaneous.

Numerous therapeutic and Prevent recommendations, with different classes of drug, for neuropathic pain have been proposed. On the basis of a systematic review and meta-analysis of all drug studies reported on since 1966, including unpublished trials, pregabalin (a GABA analogue), gabapentin (a GABA inhibitor), duloxetine (a serotonin-noradrenaline reuptake inhibitor) and various tricyclic antidepressants have strong recommendations for use and are recommended as first-line treatments for peripheral and central neuropathic pain. High-concentration capsaicin (the active component of chili peppers) patches, lidocaine patches and tramadol (an opioid with serotonin and noradrenaline reuptake inhibition effects) have weak evidence in support of their use and are recommended as second-line treatments for peripheral neuropathic pain only. Strong opioids and botulinum toxin A (administered by specialists) have weak recommendations for use as third-line treatments. However, most of these treatments have moderate efficacy based on the number needed to treat for obtaining 50% of pain relief. Furthermore, pharmacological treatments for chronic neuropathic pain are effective in <50% of patients and may be associated with adverse effects that limit their clinical utility. it is because of the much dissatisfaction with the therapy that many studies have been conducted to find the right remedy for neuropathic pain. it is on this basis that we conducted research on one of the drugs, namely estrogen. we looked at how the estrogen effects to prevent neuropathic pain.11, 14.

Estrogens are reported to reduce the sensation of pain and estrogen acting on beta receptors can provide protection from pain due to inflammation. In neuropathic pain, estrogen can modulate pain via both peripheral and central routes. In the peripheral nerves, especially in the dorsal horn of the spinal cord and DRG (dorsal root ganglia) through non-genomic, genomic, and paracrine regulation. Estrogen plays a role in the survival and regeneration of spinal neurons. In DRG neurons and spinal horn dorsal horn, estrogen reduces the entry of Ca2 + ions triggered by ATP via α estrogen receptors and selectively acts to modulate impulses mediated by P2X3 receptors. Centrally, estrogen triggers neurochemical changes that can modulate pain.11,14.

### 4.3 P2X3 receptor expression with immunohistochemistry technique

Neuropathic pain is minimally formed on the 7th postoperative day, so the administration of therapy is sufficient to be done seven days later, and the 15th day a spinal cord tissue is taken because this study is observing P2X3 receptor expression after administration of therapy, did not observe therapeutic maintenance.

This study conducted observations on the medial spinal cord, especially in the posterior horn, because P2X3 receptors are highly expressed in mammals and human CNS, especially in the posterior horn, spinal cord, and dorsal root ganglion (DRG). Additionally, the posterior horn of the spinal cord is also the first relay station for nociceptive information from the peripheral to the brain.

Observation of P2X3 receptor expression is done by indirect immunohistochemistry techniques with BSA method because P2X3 receptors can bind to monoclonal antibodies on immunohistochemistry staining, so they can be evaluated. This method uses two kinds of antibodies, which are primary (unlabeled) antibodies and secondary (unlabeled) antibodies. The primary antibody is in charge of recognizing the antigen identified in the tissue (first layer), while the secondary antibody will bind to the primary antibody (second layer). Secondary antibodies are primary anti-antibodies.

Based on the analysis research, it shows the activation of P2X3 receptors in a state of neuropathic pain. This could be seen in the placebo group who experienced neuropathic pain, describing a significantly higher percentage of P2X3 receptor expression compared to the negative control group, which did not experience neuropathic pain. Several studies have reported the activation of P2X3 receptors in the posterior horn of the spinal cord in the development and maintenance of neuropathic pain due to nerve lesions. P2X3 receptor activation plays an important role in the onset of central sensitization and wind-up in conditions of chronic pain [14,24].

Based on the results of the study, there is a decrease in P2X3 expression in the group receiving therapy compared to the positive control group with a significant difference. This shows that Estrogen can block P2X3 receptor ion channel and decrease P2X3 receptor activity, blocking calcium influx when P2X3 receptor channel is activated by ATP so that plasticity in nerve cells does not occur and neuropathic pain can be inhibited [14]. From this study, it was found how the role of estrogen can prevent neuropathic pain, where is estrogen could decrease the expression of P2X3 receptors and prolonged the onset of thermal hyperalgesia. So, both of these explained that estrogen has a role in preventing the occurrence of neuropathic pain after peripheral nerve lesions. The limitation of this study is that the research was carried out on experimental animals so that it needs to be continued in the future with clinical research involving others biomarkers for neuropathic pain.

## 5 Conclusion

Estrogen able to cut P2X3 receptor expression and prolong the onset of thermal hyperalgesia, so both of them explain that estrogen has a role in preventing neuropathic pain after peripheral nerve lesions.

## Author contribution

RHA, BAM, NUW, NAA, UQI and AGT wrote the manuscript and participated in the study design. RHA, BAM, NUW, NAA, UQI and AGT drafted and revised the manuscript. RHA, NUW, NAA, and UQI performed head trauma treatment and surgery. RHA, NAA, UQI, and RZ performed bioinformatics analyses and revised the manuscript. All authors read and approved the final manuscript.

## Registration of research studies

None.

## Guarantor

Rohadi Muhammad Rosyidi.

## Funding

No funding or sponsorship.

## Ethical approval

All procedure for Animal experiment has been approved by Animal Care and Use Committee (ACUC) Faculty of Veterinary Medicine, Airlangga University, Number: 236-KE.

## Consent

This manuscript does not involve human participants, human data, or human tissue.

## Provenance and peer review

Not commissioned, externally peer reviewed.

## Declaration of competing interest

The authors declare that they have no conflict of interests.
